# AMDORAP: Non-targeted metabolic profiling based on high-resolution LC-MS

**DOI:** 10.1186/1471-2105-12-259

**Published:** 2011-06-24

**Authors:** Hiroki Takahashi, Takuya Morimoto, Naotake Ogasawara, Shigehiko Kanaya

**Affiliations:** 1 Graduate School of Information Science, Nara Institute of Science and Technology, 8916-5 Takayama, Ikoma, Nara 630-0192, Japan; 2 Biological Science Laboratories, Kao Corporation, 2606 Akabane, Ichikai, Haga, Tochigi 321-3497, Japan

## Abstract

**Background:**

Liquid chromatography-mass spectrometry (LC-MS) utilizing the high-resolution power of an orbitrap is an important analytical technique for both metabolomics and proteomics. Most important feature of the orbitrap is excellent mass accuracy. Thus, it is necessary to convert raw data to accurate and reliable *m/z *values for metabolic fingerprinting by high-resolution LC-MS.

**Results:**

In the present study, we developed a novel, easy-to-use and straightforward *m/z *detection method, AMDORAP. For assessing the performance, we used real biological samples, *Bacillus subtilis *strains 168 and MGB874, in the positive mode by LC-orbitrap. For 14 identified compounds by measuring the authentic compounds, we compared obtained *m/z *values with other LC-MS processing tools. The errors by AMDORAP were distributed within ±3 ppm and showed the best performance in *m/z *value accuracy.

**Conclusions:**

Our method can detect *m/z *values of biological samples much more accurately than other LC-MS analysis tools. AMDORAP allows us to address the relationships between biological effects and cellular metabolites based on accurate *m/z *values. Obtaining the accurate *m/z *values from raw data should be indispensable as a starting point for comparative LC-orbitrap analysis. AMDORAP is freely available under an open-source license at http://amdorap.sourceforge.net/.

## Background

Metabolomics is defined as technology designed to give us the broadest, least biased insight into the richly diverse population of small molecules present in living things [1]. Understanding cells at the levels of the transcriptome and metabolome provides insight into the network of complex biological regulations [2-5]. Metabolites within cells have the diverse range of chemical and physical properties and the wide range of those concentrations [6]. To achieve metabolomics, two analytical platforms, i.e., mass spectrometry (MS) and nuclear magnetic resonance spectroscopy (NMR), have been widely used [7,8]. Chromatography-MS technologies play a central role in measuring the complex biological samples. Out of these, liquid chromatography-MS (LC-MS) is capable of detecting a broader range of metabolites than other MS technologies such as gas chromatography-MS and capillary electrophoresis-MS [9]. Therefore, LC-MS has become more widely used in metabolomics analysis. An orbitrap mass analyzer is the most recent addition to the set of tools that can be applied to identification, characterization and quantitation of components in biological systems since its commercial introduction in 2005 [10]. Orbitrap-based MSs have been proven to be a powerful tool in proteomics because they have ≈100 000 resolving power at a mass-to-charge ratio (*m/z*) 400 [11,12]. The most important feature of the orbitrap is that it can stably maintain excellent mass accuracy without re-calibration, and does not require the use of calibration standards [13]. Accurate *m/z* values can be used to define molecular formulae in putative identification of metabolites [7,14]. Consequently, in the field of non-targeted metabolomics, those instruments make it possible to identify candidate molecular formulae from mass differences in measured *m/z *values [15,16].

Public databases of chemical compounds such as ChEBI [17], HMDB [18], KEGG [19], KNApSAcK [20] and PubChem [21] provide suitable compounds for each molecular formula without measuring reference samples in advance. The species-metabolite relationship database KNApSAcK, for example, can easily narrow down candidates from accurate masses according to the species information or the type of ion adducts [22,23]. Several molecular ion adducts should be considered especially when the ionization of molecules in samples is performed by electrospray ionization [24,25]. Once given, the accurate *m/z *values can lead to the information of molecular formulae and candidate compounds by considering the mass differences, the appropriate ion adduct and the species together. However, it should be noted that structural isomers and stereoisomers with the same mass require the complicated chromatographic separation before mass analyzing [7].

Allen et al. [26] analyzed several "silent mutants" of yeasts (viable mutants with no obvious phenotype) by comparing extracellular metabolites using LC-MS data collected in a non-targeted approach. In preprocessing the LC-MS data, they skipped peak detection and annotation schemes typically used for such data; instead, they reduced data into a single aggregate MS vector and applied clustering and machine learning methods. Their study demonstrated the effectiveness of metabolic fingerprinting of extracellular extracts by non-complicated preprocessed data. Metabolic fingerprinting with the exclusion of *m/z *resolution, however, is impossible to get more insight from same data sets. The high-resolution of the orbitrap can be exploited in metabolic fingerprinting. In NMR or Fourier transform ion cyclotron resonance based MS (FT-ICR-MS), valid information about metabolic regulation in biological samples can be obtained by resolving power alone, even without any chromatographic separation [27].

An easy-to-use, flexible and automated tool is a key to success in metabolomics studies. This is particularly the case in high-resolution MS analyses mainly because of the data size. Our aim is to estimate more accurate *m/z *values and extract interesting *m/z *values from raw data in comparative LC-orbitrap analysis. In the present study, we describe a novel straightforward *m/z *detection method, "AMDORAP" (**A**ccurate **m**/z **d**etection method for LC-**o**rbit**rap**) for high-resolution MS (e.g., the orbitrap) by taking advantage of its stable mass accuracy.

## Implementation

Several freely available frameworks for analyzing LC-MS data sets have been developed [28]. The typical MS data processing workflow comprises multiple stages, including filtering, feature detection, alignment and normalization. In MZmine 2 [29,30], peak alignment across samples, for example, follows peak detection for individual samples. The Bioconductor package XCMS [31,32] mainly consists of peak detection, peak matching and retention time alignment. A common concept shared by widely used methods, including MZmine 2 and XCMS, is that peak detection step for both *m/z *and retention time dimensions is executed for an individual sample, or scan, followed by an alignment (or merging) step across samples. The most important reason for using high-resolution MS is to obtain more accurate *m/z *values from biological samples. That makes it possible to identify correct candidate molecular formulae from mass differences alone. Since the orbitrap can determine *m/z *values extremely accurately, we assumed that *m/z *values derived from compounds with the same compositional formula, including structural isomers and stereoisomers, should be robust with respect to retention time and differences between samples.

**Figure 1:**
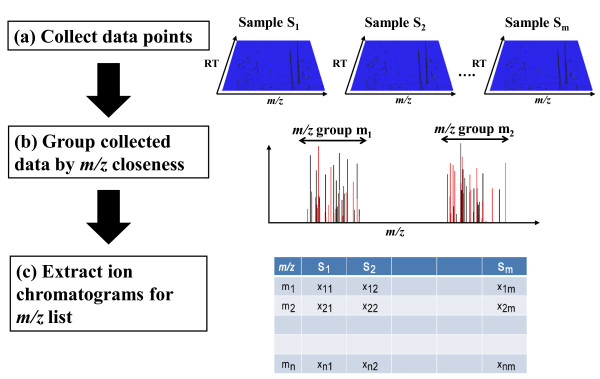
**Illustration of AMDORAP outline**. AMDORAP method consists of three steps. **(a) **Collect data points. **(b) **Group collected data by *m/z *closeness. **(c) **Extraction chromatograms for *m/z *list.

In this study, we developed the preprocessing method, AMDORAP (**A**ccurate **m**/z **d**etection method for LC-**o**rbit**rap**) written in the R programming language [33] in order to attain the quick comparison of metabolic profiling by high-resolution MS. Figure 1 illustrates the AMDORAP procedure, which comprises three steps:

1. Collect data points with intensities larger than a threshold for all samples.

2. Group collected data points by *m/z *closeness, and estimate representative *m/z *values for individual *m/z *groups.

3. Extract ion chromatograms for the *m/z *list.

The main idea motivating this procedure is that peak picking and alignment steps of *m/z *values should be performed in a single step. In the following section, the AMDORAP performance was assessed using data sets in the positive mode from two *Bacillus subtilis *strains 168 and MGB874 [34].

## Results and Discussion

### Sample preparation and experimental conditions

In order to assess the AMDORAP performance, we performed the experiments and then prepared the biological data sets. Two *Bacillus subtilis *strains, wild-type 168 and the genome reduced strain MGB874 [34], were used for metabolome analysis. The cells were cultured at 37°C to an OD_600 _value of 4.0 in the early stationary phase of growth, in Spizizen's minimal medium (SMM) [35] supplemented with 0.5% glucose, 5 *μ*g/ml tryptophan, 20 *μ*g/ml methionine and trace elements [36]. Metabolite extraction was performed according to Takahashi et al. [23]. The culture media were passed through a 0.4 *μ*m HTTP filter (Millipore). Residual cells on the filter were washed twice with HPLC grade water and then immersed in 2 ml of methanol. After incubation at 4°C overnight, the extracts were centrifuged at 9000 × *g *at 4°C for 10 min, filtered through 0.2 *μ*m PTFE membrane (Advantec), evaporated at room temperature and stored at -80°C. The extracts were dissolved in 200 *μ*l of 80% methanol before analysis in the LC-orbitrap.

Mass analysis was performed using a Paradigm MS4 system (Michrome BioResources) coupled to an LTQ-orbitrap XL-HTC-PAL system (Thermo Fisher Scientific). All experimental events were controlled by Xcalibur software version 2.0.7 (Thermo Fisher Scientific). HPLC was performed under the conditions as described by Iijima et al. [37]. Samples were injected into to a TSKgel column ODS-100V (4.6 × 250 mm, 5 *μ*m; TOSOH). Water (HPLC grade; solvent A) and acetonitrile (HPLC grade; solvent B) were used as the mobile phase with 0.1% v/v formic acid. The gradient program was as follows: 3% B to 97% B (45 min), 97% B (5 min) and 10% B (10 min). The flow rate was set to 0.5 ml/min. The ESI setting was as follows: spray voltage 4.5 kV and capillary temperature 350°C for the positive ionization mode. Nitrogen sheath gas and auxiliary gas were set at 60 and 20 arbitrary units, respectively. A full MS scan was performed in the *m/z *range 70-1500 at a resolution of 60 000. Simultaneously, top three MS^2 ^spectra within each full MS scan were gained by the linear ion trap at a collision energy of 35 eV. Thermo Fisher mass spectrometry RAW files were converted from profile mode into centroid mode using the ReAdW program [38].

### AMDORAP performance

#### Collection of data points

**Figure 2:**
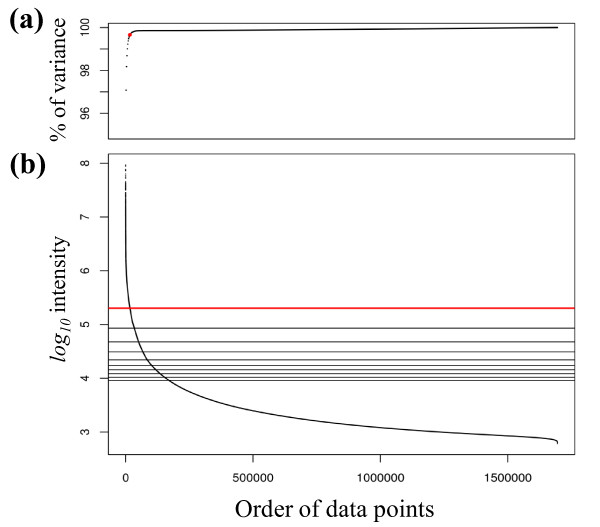
**Intensity distribution of LC-orbitrap data**. *B. subtilis *strain 168 was measured using the LC-orbitrap in the positive mode. The centroid data has 1 694 959 data points, obtained with 1945 scans over 45 minutes (data size is 29 MB). **(a) **% of total variance. Each dot corresponds to the percent of variance explained by each corresponding percent of the data from 0.1-100 at interval of 0.1%. Red dot corresponds to the top 1% of the data, which explained 99.7% of the total variance. **(b) **Intensity distribution. All data points are plotted. Nine black horizontal solid lines correspond to 90^th^- 98^th ^percentile values at interval of 1%, and the red line corresponds to the 99^th ^percentile value.

**Figure 3:**
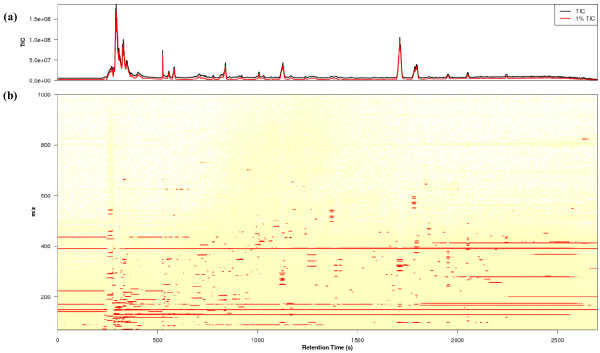
**All data vs Top 1% of data**. **(a) **The comparison of the total ion chromatograms of all and top 1% of data. The abscissa and ordinate axes correspond to the retention times and ion intensity, respectively. The total ion chromatograms of all data and top 1% of data were plotted as black and red solid lines, respectively. **(b) **Two dimensional map (*m/z *vs retention times). Top 1% of data are plotted as red points.

Figure 2b shows the intensity distribution of a centroid data from *B. subtilis *strain 168. The total number of data points was 1 694 959 (1945 scans within 45 minutes). The top 1% of the data (represented by a red dot in Figure 2a) could explain 99.7% of the total variance of all data points. Thus, almost all data obtained by the LC-orbitrap can be considered as background noise. Here, we assumed that the top 1% of the data was detected ions, and the other 99% was noise for each sample in the collecting step. Figure 3 shows the total ion chromatograms and two dimensional map. The total ion chromatogram of top 1% of data highly correlates with that of all data (in Figure 3a) and then top 1% of data is extensively scattered in both dimensions (in Figure 3b), suggesting that top 1% of data can explain the characterization of all data with respect to intensities and dimensions.

#### Grouping collected data points and estimation of representative *m/z *values for individual groups

As the second step, all collected *m/z *values are grouped by closeness, i.e., if differences between the neighboring *m*/*z *values are within 5 ppm (default setting), they are grouped together. There is no limit of data points within one *m*/*z *group as long as this constrain is fulfilled. Out of the *m/z *alignment methods, Kazmi et al. [39] developed the method to create bins and then combine consecutive bins together according to the constrains, similar to complete linkage hierarchical clustering. While they must consider the origins of *m/z *values, our method is to collect all data points with relatively higher intensities and then deal with collected data as one spectrum. Consequently, the grouping of *m/z *values is feasible in one step.

**Figure 4:**
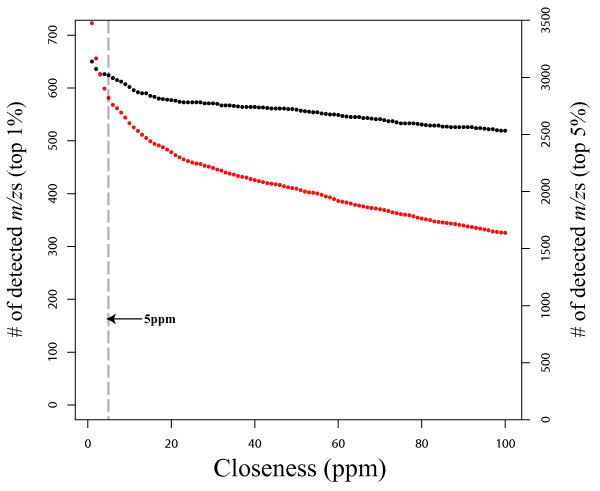
**Numbers of detected *m/z *values by the parameter of closeness**. Black (ordinate axis on the left) and red (ordinate axis on the right) dots correspond to the numbers of detected *m/z *values using 1 and 5% of the data, respectively.

Median *m/z *values of individual *m/z *groups are defined as the peak values among all samples. Figure 4 shows the relationship between closeness and the number of *m/z *groups by simultaneously using two data sets. In case of closeness 5 ppm (default setting) for the top 1 and 5% of data points, 624 (black dots in Figure 4) and 2821 (red dots) *m/z *groups were obtained, respectively. According to Werf et al. [40], the *in silico *metabolome of *B. subtilis *is covered by 537 compounds. Of those, 282 compounds are commercially available. Other compounds can not be identified by the method of measuring authentic compounds. Additionally, Pluskal et al. [41] and Iijima et al. [37], for example, identified 123 metabolites from approximately 1900 peaks in yeast and at most 29 metabolites identified by comparison with authentic compounds (they called grade A) from ~4700 peaks in tomato, respectively. Those studies indicate that most of obtained peaks from LC-MS data would remain unknown even after peak detection. We concluded that 624 *m/z *groups could be sufficient to express the cell state as starting point for LC-orbitrap analysis.

For identification of the ions by MS^2 ^data, we made an in-house database for *B. subtilis *compounds by using KEGG database. All reactions associated with *B. subtilis *were extracted and 890 compounds were set to be as the database (Additional file 1). After database search ([M+H]^+^) within ±5 ppm for MS^2 ^precursor *m/zs *in two *B. subtilis *data, 20 available authentic compounds (Additional file 2) were measured under the same conditions for *B. subtilis *strains. Out of limited MS^2 ^spectra in *B. subtilis *samples, 14 compounds were manually identified by measuring the authentic compounds. Next, we performed a comparison study for *m/z *accuracy between AMDORAP, MZmine 2 and XCMS. The steps including Chromatogram builder (*m/z *tolerance = 0.01), RANSAC aligner and Peak finder were performed by MZmine 2. In XCMS parameters for UPLC-orbitrap data, Dunn et al. [6] showed that two parameters, *snthresh *and *bw*, significantly affected the processed data, e.g., the number of peaks detected and the peak area reproducibility. For XCMS, the parameters were set to be "centWave", *bw *= 60, *snthresh *= 2, *ppm *= 3 and *mzwid *= 0.02 with all other default settings. Table 1 summarizes the comparisons of observed *m*/*z *values associated with 14 identified compounds. Seven *m/z *values obtained by AMDORAP were closest to the theoretical masses. While all errors of observed *m/z *values in AMDORAP were distributed within ±3 ppm, some errors in MZmine 2 and XCMS were over ±100 ppm, e.g., tryptophan, uridine and glutamine, suggesting that our procedure can detect more accurate *m/z *values than others. In the case of other parameter settings for XCMS, a few compounds were not detected (data not shown). In compound searches using mass differences alone, *m/z *values with errors over ±100 ppm could be no longer correctly annotated by leveraging the high-resolution power of the orbitrap. This comparison shows that our method has the best performance in detecting accurate *m/z *values and can allow us to identify correct candidate compounds by mass differences alone. According to Goerlach et al. [25], 30 and 14 different types of molecular ion adducts exist in the positive and negative modes, respectively. Furthermore, structural isomers and stereoisomers have the same mass. Therefore, it should be noted that putative identification of metabolites based on the accurate *m/z *values is carefully performed to avoid the misleading results.

**Table 1:** Comparison of detected *m/z *values for fourteen compounds by AMDORAP, MZmine 2 and XCMS

			AMDORAP		MZmine 2		XCMS	
metabolite	formula	**theoretical** [M + H]^+^	observed *m/z*	error ppm	observed *m/z*	error ppm	Observed *m/z*	error ppm
serine	C_3_H_7_NO_3_	106.04987	**106.04960**	**-2.55**	106.04955	-3.02	106.04958	-2.68
valine	C_5_H_11_NO_2_	118.08626	**118.08604**	**-1.78**	118.08602	-2.01	118.08422	-17.26
glutamine	C_5_H_10_N_2_O_3_	147.07642	147.07666	1.64	**147.07642**	**0.03**	147.14403	459.68
lysine	C_6_H_14_N_2_O_2_	147.11280	**147.11277**	**-0.23**	147.11067	-14.49	147.10871	-27.82
glutamic acid	C_5_H_9_NO_4_	148.06043	148.06055	0.76	**148.06042**	**-0.09**	148.05154	-60.10
methionine	C_5_H_11_NO_2_S	150.05833	150.05817	-1.06	**150.05818**	**-0.96**	150.04993	-55.93
D-alanyl-D-alanine	C_6_H_12_N_2_O_3_	161.09207	161.09181	-1.59	**161.09197**	**-0.60**	161.08698	-31.61
phenylalanine	C_9_H_11_NO_2_	166.08626	166.08617	-0.53	**166.08618**	**-0.44**	166.08285	-20.50
citrulline	C_6_H_13_N_3_O_3_	176.10297	176.10280	-0.96	**176.10281**	**-0.87**	176.11662	77.51
tyrosine	C_9_H_11_NO_3_	182.08117	182.08113	-0.21	182.08110	-0.41	**182.08119**	**0.09**
tryptophan	C_11_H_12_N_2_O_2_	205.09715	**205.09732**	**0.81**	205.06284	-167.29	205.09010	-34.38
pantothenate	C_9_H_17_NO_5_	220.11795	**220.11798**	**0.14**	220.11414	-17.29	220.12327	24.18
uridine	C_9_H_12_N_2_O_6_	245.07681	**245.07692**	**0.44**	245.10884	130.69	245.08308	25.58
methylthioadenosine	C_11_H_15_N_5_O_3_S	298.09684	**298.09708**	**0.80**	298.08231	-48.72	298.09719	1.18

Comparison of detected *m/z *values between AMDORAP, MZmine 2 and XCMS. Seven *m/z *values obtained by AMDORAP, i.e., serine, valine, lysine, tryptophan, pantothenate, uridine and methylthioadenosine, were closest to the theoretical masses.

#### Extraction of ion chromatograms for the *m/z *list

**Figure 5:**
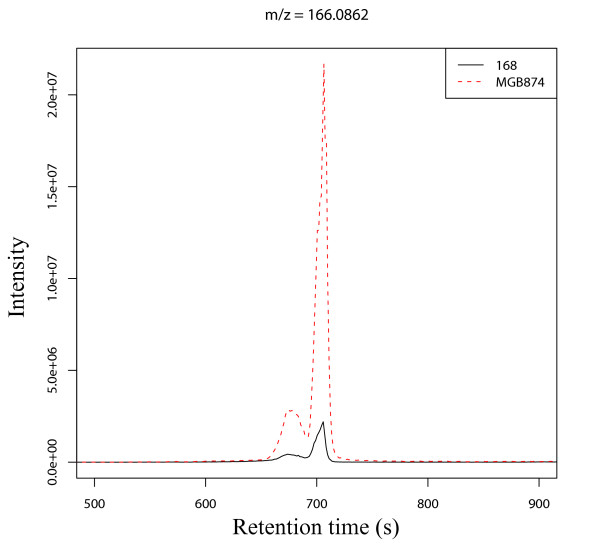
**A chromatogram trace of phenylalanine**. Chromatogram trace of *m/z *slice 166.08534-166.08700 is presented. Black solid and red dashed lines correspond to *B. subtilis *strains 168 and MGB874, respectively. Two peak areas were also observed for an authentic compound under our conditions.

The final step is to extract ion chromatograms for the *m/z *list within ±5 ppm (default setting). AMDORAP provides two types of representative values for detected *m/z *values. One is the sum of total ion chromatogram and another is the sum of selected peak area by a signal-to-noise ratio cutoff for Gaussian filtered chromatogram [42]. Of 624 *m/z *values, 603 reliable chromatograms were extracted by manually checking. We judged the chromatograms with noisy baseline or stretched across the experimental time, i.e., 45 min, as unreliable chromatograms in this study. Additional file 3 shows 21 extracted ion chromatograms judged to be unreliable chromatograms. The numbers of chromatograms with only one peak through 45 minutes, were 471 (79%) and 453 (75%) in *B. subtilis *strains 168 and MGB874, respectively; the numbers of chromatograms with two peaks were 86 (14%) and 113 (18%). As showing in Figure 5, two peaks were seen in a chromatogram of phenylalanine; this phenomenon was confirmed under our experimental conditions by measuring the authentic phenylalanine, indicating that some of the chromatograms with two peaks originate from unique compounds. Those results suggest that to separate the peaks by the retention time could mislead the identification of the ions and clues about the chemical structures corresponding to those peaks could be obtained without separating chromatograms by the retention time. Hence, almost all chromatograms based on AMDORAP could be identified as unique compounds even without separation of identical *m*/*z *peaks by the retention time. Taken together, the reliable *m/z *grouping process is sufficient for comparison of metabolic fingerprinting based on high-resolution LC-MSs.

## Conclusions

In metabolic profiling by the high-resolution mass technologies, it is important to convert raw data to reliable *m/z *values in order to quickly get the information of correct candidate metabolites in biological samples. With respect to obtained *m/z *accuracy, comparison study was performed using only 14 identified compounds. Clearly, the *m/z *errors by AMDORAP are smallest, although the number of compared compounds might be not enough. In the range of tested parameters, we couldn't get better results for 14 compounds by MZmine 2 and XCMS. This suggests that parameter optimization of those tools is time consuming process and difficult to find out best settings for both dimensions, i.e., *m/z *and retention time. Furthermore, it would suggest that both mass and retention time alignment processes introduce the larger errors for obtained *m/z *values, while AMDORAP uses only the ions with relatively higher intensities for estimating the *m/z *values. In addition, a signal-to-noise ratio cutoff by Gaussian filtering could allow us to achieve a reliable comparison of the ion abundances between samples, even when there are peaks with noisy baseline. Thus, AMDORAP can detect more accurate *m/z *values from raw data and provide the platform for metabolic fingerprinting. Information of MS*^n^*, retention time and behaviors of the authentic compounds has the essential roles to finally verify the ions as particular metabolites. However, the extraction of interesting accurate *m/z *values by AMDORAP should be indispensable as a starting point for comparative LC-orbitrap analysis, because of the limitations of available authentic compounds and simultaneously obtained MS^2 ^spectra with a full MS scan per sample.

## Competing interests

The authors declare that they have no competing interests.

## Availability and requirements

**Project name: **AMDORAP

**Project home page: **http://amdorap.sourceforge.net/

**Operating systems: **Platform independent

**Programming language: **R

**License: **GPL v2

**Any restrictions to use by non-academics: **No

## Authors' contributions

HT performed experimental parts, implemented the methods, analyzed data sets and wrote the manuscript. TM performed extraction of metabolites. NO and SK supervised this project. All authors have read and approved the final manuscript.

## Supplementary Material

Additional file 1 **A list of 890 compounds**. This list contains 890 compounds associated with all reactions in *B. subtilis *of KEGG. Click here for file

Additional file 2 **A list of 20 authentic compounds**. These compounds were measured by LC-orbitrap. Obtained information (MS^2 ^and retention time) were used to identify the compounds. Click here for file

Additional file 3 21 **unreliable chromatograms**. 21 extracted ion chromatograms judged to be unreliable chromatograms are shown. The abscissa and ordinate axes correspond to the retention times and ion intensity, respectively. Click here for file
