# Supplementary figures and images for: AMDORAP: Non-targeted metabolic profiling based on high-resolution LC-MS
Source: BMC Bioinformatics. 2011 Jun 24;12:259. doi: 10.1186/1471-2105-12-259 (PMC3149581; doi:10.1186/1471-2105-12-259)

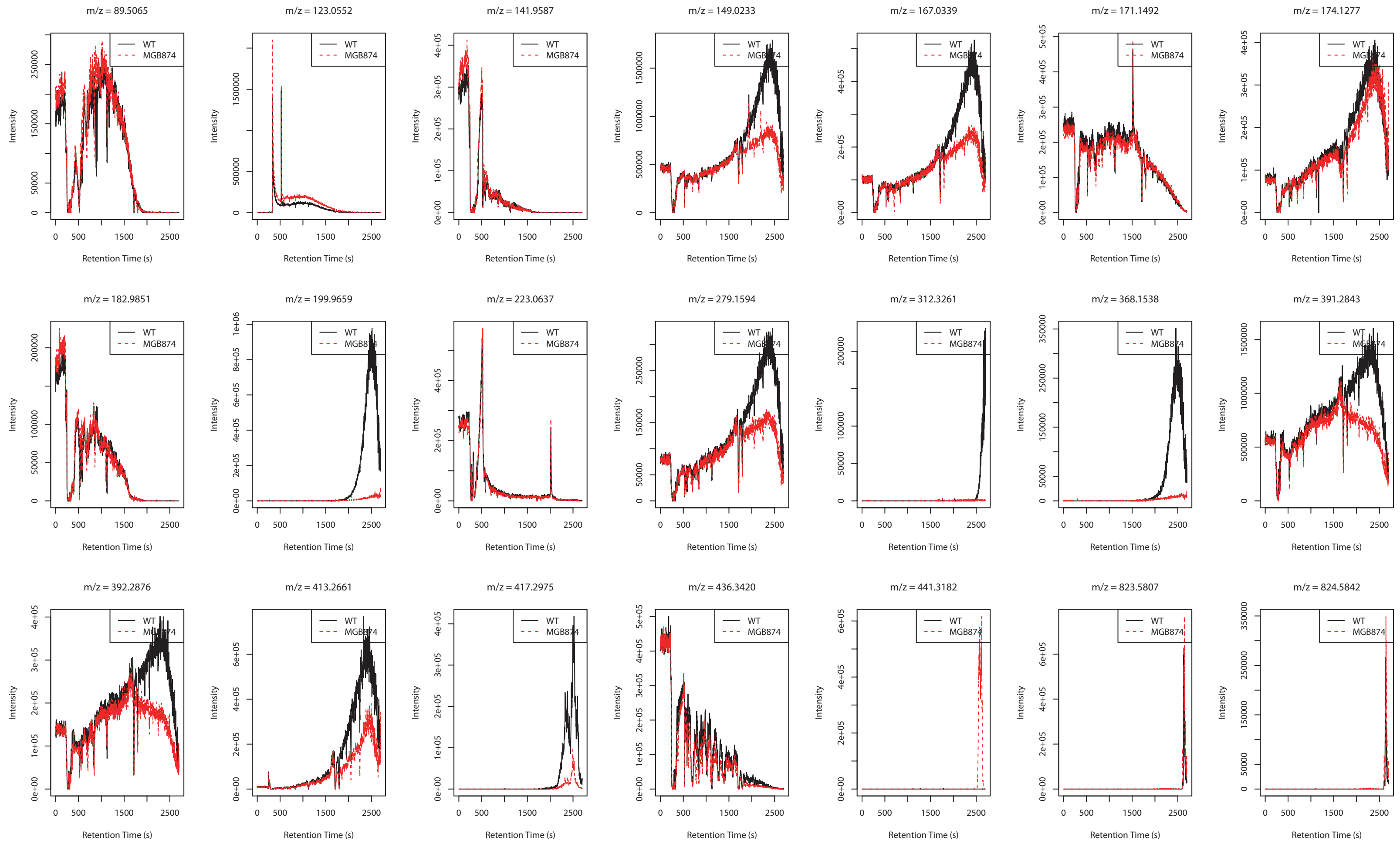

Supplement: Additional file 3 — 21 unreliable chromatograms. 21 extracted ion chromatograms judged to be unreliable chromatograms are shown. The abscissa and ordinate axes correspond to the retention times and ion intensity, respectively. [file 1471-2105-12-259-S3.PDF]
